# Replication of *EPHA1 *and *CD33 *associations with late-onset Alzheimer's disease: a multi-centre case-control study

**DOI:** 10.1186/1750-1326-6-54

**Published:** 2011-07-28

**Authors:** Minerva M Carrasquillo, Olivia Belbin, Talisha A Hunter, Li Ma, Gina D Bisceglio, Fanggeng Zou, Julia E Crook, V Shane Pankratz, Sigrid B Sando, Jan O Aasly, Maria Barcikowska, Zbigniew K Wszolek, Dennis W Dickson, Neill R Graff-Radford, Ronald C Petersen, Peter Passmore, Kevin Morgan, Steven G Younkin

**Affiliations:** 1 Department of Neuroscience, Mayo Clinic College of Medicine, Jacksonville, FL 32224, USA; 2 School of Molecular Medical Sciences, Institute of Genetics, Queens's Medical Centre, University of Nottingham, NG7 2UH, UK; 3 Biostatistics Unit, Mayo Clinic College of Medicine, Jacksonville, FL 32224, USA; 4 Division of Biomedical Statistics and Informatics, Mayo Clinic College of Medicine, Rochester, MN 55905, USA; 5 Department of Neurology, St. Olav's Hospital, Edvard Griegs Gate 8, 7006 Trondheim, Norway; 6 Department of Neuroscience, Norwegian University of Science and Technology, NTNU, 7491 Trondheim, Norway; 7 Department of Neurodegenerative Disorders, Medical Research Centre, Polish Academy of Sciences, Warsaw, Poland; 8 Department of Neurology, Mayo Clinic College of Medicine, Jacksonville, FL 32224, USA; 9 Department of Neurology, Mayo Clinic College of Medicine, Rochester, MN 55905, USA; 10 Mayo Alzheimer Disease Research Center, Mayo Clinic College of Medicine, Rochester, MN 55905, USA; 11 Centre for Public Health, School of Medicine, Dentistry and Biomedical Sciences, Queen's University Belfast, Northern Ireland, BT7 1NN, UK; 12 OB is now affiliated to Hospital de la Santa Creu i Sant Pau, 08025, Barcelona, Spain

## Abstract

**Background:**

A recently published genome-wide association study (GWAS) of late-onset Alzheimer's disease (LOAD) revealed genome-wide significant association of variants in or near *MS4A4A, CD2AP, EPHA1 *and *CD33*. Meta-analyses of this and a previously published GWAS revealed significant association at *ABCA7 *and *MS4A*, independent evidence for association of *CD2AP, CD33 *and *EPHA1 *and an opposing yet significant association of a variant near *ARID5B*. In this study, we genotyped five variants (in or near *CD2AP, EPHA1, ARID5B*, and *CD33*) in a large (2,634 LOAD, 4,201 controls), independent dataset comprising six case-control series from the USA and Europe. We performed meta-analyses of the association of these variants with LOAD and tested for association using logistic regression adjusted by age-at-diagnosis, gender, and *APOE ε4 *dosage.

**Results:**

We found no significant evidence of series heterogeneity. Associations with LOAD were successfully replicated for *EPHA1 *(rs11767557; OR = 0.87, p = 5 × 10^-4^) and *CD33 *(rs3865444; OR = 0.92, p = 0.049), with odds ratios comparable to those previously reported. Although the two *ARID5B *variants (rs2588969 and rs494288) showed significant association with LOAD in meta-analysis of our dataset (p = 0.046 and 0.008, respectively), the associations did not survive adjustment for covariates (p = 0.30 and 0.11, respectively). We had insufficient evidence in our data to support the association of the *CD2AP *variant (rs9349407, p = 0.56).

**Conclusions:**

Our data overwhelmingly support the association of *EPHA1 *and *CD33 *variants with LOAD risk: addition of our data to the results previously reported (total n > 42,000) increased the strength of evidence for these variants, providing impressive p-values of 2.1 × 10^-15 ^(*EPHA1*) and 1.8 × 10^-13 ^(*CD33*).

## Background

Following the identification of the *APOE *ε4 allele as a risk factor for late-onset Alzheimer's disease (LOAD) in 1993 [1], consistent replication of subsequently identified candidates was not achieved until 2009, when two genome-wide association studies (GWAS) [2,3] identified associations of variants in or near *CLU, PICALM *, and *CR1 *with LOAD, which were consistently replicated in multiple large, independent case-control studies [4-17]. Subsequently, a variant near *BIN1 *was reported [4] to achieve genome-wide significant association in a later GWAS published in 2010 that also replicated well in follow-up studies [14-19]. These results demonstrate the utility of the hypothesis-free GWAS approach for identifying loci that associate with LOAD and the necessity of pooling samples and data from multiple centers to obtain resources with sufficient statistical power (GWAS typically > 14,000, follow-up typically total > 28,000) to detect the modest ORs (e.g. 0.8/1.2) associated with these variants in GWAS and follow-up studies.

Two recently published companion studies by Hollingworth *et al*. [20] and Naj *et al*. [17] performed meta-analysis of two large GWAS datasets (n > 75,000). Besides *APOE, CLU, PICALM*, and *CR1*, the meta-analyses revealed association at *ABCA7 *(p = 5 × 10^-21^), *MS4A6A *(p = 1.2 × 10^-16^), *MS4A4E *(p = 1.1 × 10^-10^), *EPHA1 *(p = 6 × 10^-10^), *CD2AP *(p = 8.6 × 10^-9^) and *CD33 *(p = 1.6 × 10^-9^). In addition, the two datasets revealed opposing association (Naj *et al*. OR = 0.93, p = 0.001; Hollingworth *et al*. OR = 1.06, p = 0.03) of the variant near *ARID5B *(rs2588969) with LOAD, suggesting potential heterogeneity at this locus. In this study, we genotyped the variants identified at the *CD2AP, EPHA1*, and *CD33 *loci in our independent case-control dataset comprising six case-control series (n = 6,835). To assess the opposing associations at the *ARID5B *locus, we also genotyped the two *ARID5B *variants included in the Hollingworth *et al*. study. Genotypes from our follow-up case-control series (Mayo 2) for variants in *ABCA7, MS4A6A *and *MA4A4E *were included in Stage 3 of the Hollingworth *et al*. study, so we have not included these three variants in this study. We have performed meta-analyses of five variants (at *CD2AP, EPHA1, ARID5B *and *CD33 *loci) in our six case-control series, which showed no significant series heterogeneity. Furthermore, we have performed logistic regression analysis of our pooled series adjusting for covariates. Finally, we have used a Fisher's combined test to evaluate the significance of the association of these five variants in our data combined with the data in the Hollingworth *et al*. and Naj *et al*. studies.

## Results

We genotyped five variants (*CD2AP*; rs9349407, *EPHA1*; rs11767557, *ARID5B*; rs2588969 and rs4948288, *CD33*; rs3865444) in our independent follow-up case-control series (Mayo2) from three North American and three European Caucasian series. Detailed information about these samples is shown in Table 1 and genotype counts are shown in Table 2. Samples used in this study do not overlap with those included in the Naj *et al*. study and have not been included in any of the published LOAD GWAS. The Mayo2 dataset included in the Hollingworth *et al*. publication only included genotypes for *ABCA7, MS4A6A *and *MA4A4E*.

**Table 1:** Details of the Mayo2 samples used in this study and genotype counts

	Number of samples			Mean Age (SD)		% Female		% ε4+	
Series	AD	CON	Total	AD	CON	AD	CON	AD	CON
Jacksonville	507	967	1,474	80.0 (6.7)	81.7 (7.6)	61.9	56.3	60.2	21.8
Rochester	317	1,638	1,955	85.8 (4.5)	80.3 (5.2)	62.1	54.6	42.3	22.4
Autopsy	312	102	414	87.4 (4.8)	86.0 (4.3)	67.6	52.0	61.2	14.7
Norway	346	555	901	80.2 (7.3)	75.3 (6.8)	69.9	59.8	63.0	24.1
Poland	483	188	671	76.7 (4.8)	73.0 (5.9)	66.3	76.6	56.4	19.0
ARUK	669	751	1,420	75.6 (8.2)	76.2 (7.3)	55.6	49.9	58.0	24.4

The number of LOAD patients (AD) and controls (CON), mean age-at-diagnosis, percentage that are female and percentage that possess at least one copy of the *APOE ε *4 allele are given for each individual series. Mean age is given as age at diagnosis/entry with the standard deviation (SD) from the mean in parentheses. None of the samples comprising the Jacksonville, Rochester and autopsy-confirmed Mayo Clinic or ARUK series (comprising Bristol, Leeds, Manchester, Nottingham, Oxford and Southampton), which were included in this follow-up study overlap with those used in the Naj *et al*. study and have not been included in any of the published LOAD GWAS. The Mayo2 dataset included in the Hollingworth *et al*. publication only included genotypes for *ABCA7, MS4A6A *and *MA4A4E*.

**Table 2:** Genotype counts for each of the six Mayo2 series

	*CD2AP *(rs9349407)		*EPHA1 *(rs11767557)		*ARID5B *(rs2588969)		*ARID5B *(rs4948288)		*CD33 *(rs3865444)	
	GG/GC/CC	GG/GC/CC	TT/TC/CC	TT/TC/CC	CC/CA/AA	CC/CA/AA	GG/GA/AA	GG/GA/AA	CC/CA/AA	CC/CA/AA
Series	AD	CON	AD	CON	AD	CON	AD	CON	AD	CON
Jacksonville	254/197/41	497/369/56	339/143/19	612/301/44	188/226/81	379/400/149	164/233/99	351/426/148	251/200/41	446/386/88
Rochester	170/126/17	843/640/117	198/102/9	985/518/69	100/159/48	623/755/226	92/172/50	581/748/250	148/134/30	715/692/170
Autopsy	156/110/19	49/44/7	205/97/5	61/28/10	118/148/42	50/38/14	115/142/43	38/43/17	141/125/32	42/44/11
Norway	177/131/16	273/205/41	212/113/13	337/185/26	129/165/44	215/250/78	115/156/53	184/268/88	153/139/35	248/236/57
Poland	235/193/40	100/70/11	297/140/20	108/52/9	153/243/77	65/91/29	160/222/84	62/96/26	224/204/39	96/83/8
ARUK	341/243/55	363/317/53	386/191/20	439/234/37	236/313/101	271/367/102	208/326/122	259/351/122	289/286/67	329/307/94
Total	1333/1000/188	2125/1645/285	1637/786/86	2542/1318/195	924/1254/393	1603/1901/598	854/1251/451	1475/1932/651	1206/1088/244	1876/1748/428

The genotype counts (major allele homozygotes/heterozygotes/minor allele homozygotes) for *CD2AP *(rs9349407), *EPHA1 *(rs11767557), *ARID5B *(rs2588969 and rs4948288) and *CD33 *(rs3865444) variants are given for each individual series.

**Figure 1:**
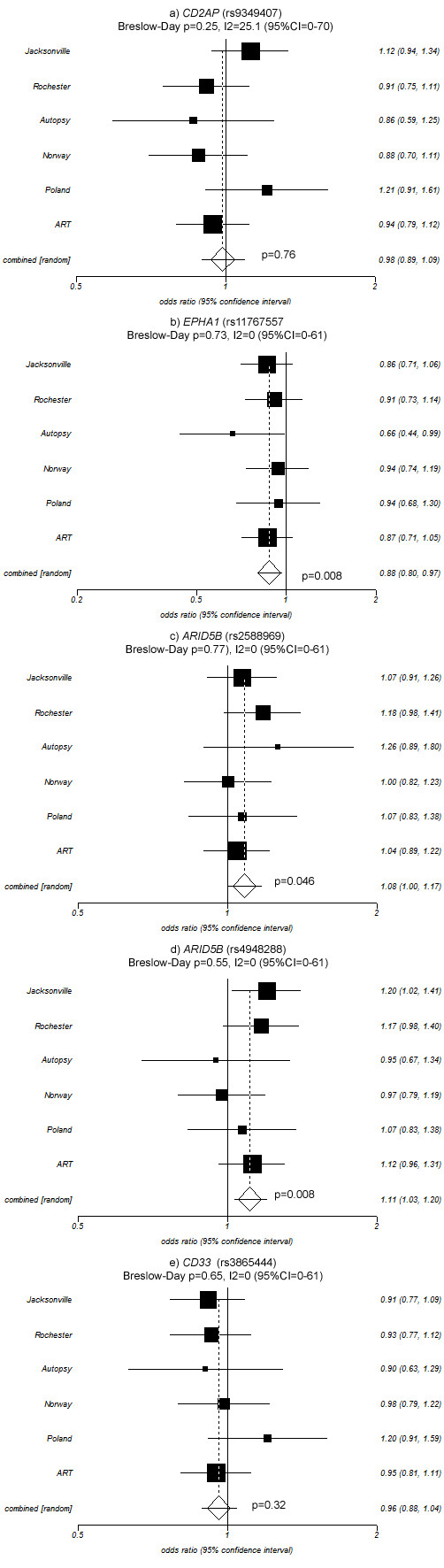
**Forest plots for meta-analysis of *CD2AP, EPHA1, ARID5B*, and *CD33 *variants in our six Mayo2 case-control series**. ORs (boxes) and 95% CI (whiskers) are plotted for each population and shown on the right of each plot. Combined OR is the overall OR calculated by the meta-analysis using a random effects model. P-values are provided for the combined ORs and Breslow-Day tests of heterogeneity. I2 gives an estimate of between studies variance.

Meta-analyses of allelic association in the six Mayo2 series performed using a DerSimonian-Laird random effects model (Figure 1) revealed a significant pooled OR for the *EPHA1 *variant (Figure 1b; OR = 0.88, p = 0.008) comparable to that previously published by Naj *et al*. (OR = 0.87) and by Hollingworth *et al*. (OR = 0.90). As shown in Figure 1c and 1d, we also observed significant association for both *ARID5B *variants (rs2588969, OR = 1.08, p = 0.046; rs4948288, OR = 1.11, p = 0.008) with ORs comparable to those reported by Hollingworth *et al*. (OR = 1.06 and 1.07, respectively) and in the opposing direction to those reported by Naj *et al*. for rs2588969 (Stage 1+2 OR = 0.93, p = 7.7 × 10^-4^). As shown in Figure 1a and 1e, we did not observe significant association for *CD2AP *(OR = 0.98, p = 0.76) or *CD33 *(OR = 0.96, p = 0.32) in our meta-analyses. Breslow-Day tests provided no significant evidence that the ORs for any of these variants were heterogeneous among our series (all p > 0.25), as shown in Figure 1. The variant with the most heterogeneity was *CD2AP *(rs9349407) where the estimated percentage of variation due to heterogeneity across studies (I^2^) was 25.1% (95% CI 0%-70%) suggesting the presence of some heterogeneity for that variant.

To adjust for important covariates, we included age-at-diagnosis/entry, sex and *APOE ε 4 *dosage in logistic regression analyses of all five variants in each of the six Mayo2 series; in our analysis of all Mayo2 series combined, series was included as an additional covariate. Table 3 shows the results for the six Mayo2 series combined (Mayo follow-up) as well as for each of the six individual Mayo2 series. For the purpose of comparison, we have also included in Table 3 the published GWAS results for the same variants. Adjustment for covariates revealed comparable ORs to those obtained in the meta-analyses, with improved p-values for the *EPHA1 *(OR = 0.87, p = 5 × 10^-4^), *CD33 *(OR = 0.92, p = 0.049) and *CD2AP *(OR = 0.97, p = 0.56) loci. However, the associations of the *ARID5B *variants were no longer significant following adjustment for covariates (rs2588969: OR = 1.05, p = 0.30, rs4948288: OR = 1.07, p = 0.11) suggesting that these associations may be dependent upon the series, age-at-diagnosis/entry, sex and/or *APOE ε 4 *dosage of the individual.

**Table 3:** Association of *CD2AP, EPHA1, ARID5B*, and *CD33 *variants with LOAD in the initial studies (ADGC and GERAD+) and Mayo2 follow-up series

	*N*^a^		MAF^b^		Association test	
Study	Cases	Controls	Cases	Controls	OR (95% CI)	p-value
***CD2AP-*rs9349407-C (minor) allele**						

ADGC Discovery (Stage 1)	8,309	7,366			1.14 (1.08-1.21)	**1.2 × 10^-6^**
ADGC Replication (Stage 2)	3,531	3,565			1.07 (0.98-1.17)	**0.12**
ADGC combined analysis (Stages 1+2)	11,840	10,931			1.12 (1.07-1.18)	**1.0 × 10^-6^**
Hollingworth *et al*. (GERAD + Consortia)	6,283	7,165			1.11 (1.04-1.18)	**8 × 10^-4^**
Mayo2^c^	2,521	4,055	0.27	0.27	0.97 (0.89-1.07)	0.56
Jacksonville	492	922	0.28	0.26	1.10 (0.91-1.33)	0.34
Rochester	313	1,600	0.26	0.27	0.88 (0.70-1.09)	0.24
Autopsy	285	100	0.26	0.29	0.98 (0.65-1.47)	0.92
Norway	324	519	0.25	0.28	0.81 (0.62-1.06)	0.13
Poland	468	181	0.29	0.25	1.04 (0.77-1.42)	0.79
ARUK	639	733	0.28	0.29	0.97 (0.81-1.16)	0.72
ADGC/Hollingworth^d^	18,123	18,096				1.2 × 10^-10^
Mayo2/ADGC/Hollingworth^e^	20,644	22,151				6.5 × 10^-11^

***EPHA1-*rs11767557-C (minor) allele**						

ADGC Discovery (Stage 1)	8,309	7,366			0.85 (0.80-0.90)	**7.3 × 10^-8^**
ADGC Replication (Stage 2)	3,531	3,565			0.94 (0.86-1.03)	0.17
ADGC combined analysis (Stages 1+2)	11,840	10,931			0.87 (0.83-0.92)	**2.4 × 10^-7^**
Hollingworth et al (GERAD + Consortia)	6,283	12,935			0.90 (0.85-0.95)	**3.4 × 10^-4^**
Mayo2^c^	2,509	4,055	0.19	0.21	0.87 (0.78-0.96)	**5.5 × 10^-4^**
Jacksonville	501	957	0.18	0.20	0.86 (0.70-1.06)	0.17
Rochester	309	1,572	0.19	0.21	0.89 (0.69-1.13)	0.33
Autopsy	307	99	0.17	0.24	0.66 (0.43-1.02)	0.06
Norway	338	548	0.21	0.22	0.94 (0.71-1.24)	0.67
Poland	457	169	0.20	0.21	0.93 (0.66-1.31)	0.67
ARUK	597	710	0.19	0.22	0.85 (0.69-1.04)	0.12
ADGC/Hollingworth^d^	18,123	18,096				4.2 × 10^-12^
Mayo2/ADGC/Hollingworth^e^	20,632	27,921				2.1 × 10^-15^

***ARID5B-*rs2588969-A (minor) allele**						

ADGC Discovery (Stage 1)	8,309	7,366			0.88 (0.84-0.93)	**1.1 × 10^-6^**
ADGC Replication (Stage 2)	3,531	3,565			1.05 (0.97-1.13)	0.23
ADGC combined analysis (Stages 1+2)	11,840	10,931			0.93 (0.89-0.97)	**0.001**
Hollingworth et al (GERAD + Consortia)	6,283	7,165			1.06 (1.01-1.13)	**0.03**
Mayo2^c^	2,571	4,102	0.40	0.38	1.05 (0.96-1.14)	0.30
Jacksonville	495	928	0.39	0.38	1.04 (0.88-1.23)	0.63
Rochester	307	1,604	0.42	0.38	1.12 (0.92-1.37)	0.26
Autopsy	308	102	0.38	0.32	1.24 (0.86-1.79)	0.24
Norway	338	543	0.37	0.37	1.05 (0.83-1.33)	0.69
Poland	473	185	0.42	0.40	0.91 (0.68-1.20)	0.49
ARUK	650	740	0.40	0.39	1.05 (0.88-1.24)	0.61
ADGC/Hollingworth^d^	18,123	18,096				7.6 × 10^-9^
Mayo2/ADGC/Hollingworth^e^	20,694	22,198				2.3 × 10^-9^

**ARID5B-rs4948288-A (minor) allele**						

ADGC Discovery (Stage 1)	8,309	7,366				
ADGC Replication (Stage 2)	3,531	3,565				
ADGC combined analysis (Stages 1+2)	11,840	10,931				
Hollingworth et al (GERAD + Consortia)	6,992	13,472			1.07 (1.03-1.15)	**3.6 × 10^-3^**
Mayo2^c^	2,556	4,058	0.42	0.40	1.07 (0.99-1.16)	0.11
Jacksonville	496	925	0.43	0.39	1.13 (0.96-1.34)	0.14
Rochester	314	1,579	0.43	0.40	1.08 (0.89-1.32)	0.43
Autopsy	300	98	0.38	0.39	0.91 (0.63-1.32)	0.61
Norway	324	540	0.40	0.41	1.06 (0.83-1.34)	0.64
Poland	466	184	0.42	0.40	0.90 (0.68-1.20)	0.48
ARUK	656	732	0.43	0.41	1.13 (0.96-1.33)	0.14
Mayo2/ADGC/Hollingworth^e^	9,548	17,530				4.0 × 10^-4^

***CD33 -*rs3865444-A (minor) allele**						

ADGC Discovery (Stage 1)	8,309	7,366			0.88 (0.84-0.93)	**8.2 × 10^-7^**
ADGC Replication (Stage 2)	3,531	3,565			0.91 (0.85-0.99)	**0.02**
ADGC combined analysis (Stages 1+2)	11,840	10,931			0.89 (0.86-0.93)	**1.1 × 10^-7^**
Hollingworth et al (GERAD + Consortia)	6,283	7,165			0.89 (0.84-0.95)	**2.2 × 10^-4^**
Mayo2^c^	2538	4052	0.31	0.32	0.92 (0.84-1.00)	**4.9 × 10^-2^**
Jacksonville	492	920	0.29	0.31	0.82 (0.68-0.98)	**0.03**
Rochester	312	1,577	0.31	0.33	0.88 (0.72-1.08)	0.23
Autopsy	298	97	0.32	0.34	0.84 (0.57-1.24)	0.39
Norway	327	541	0.32	0.32	0.89 (0.70-1.14)	0.37
Poland	467	187	0.30	0.26	1.00 (0.72-1.37)	0.99
ARUK	642	730	0.33	0.34	0.98 (0.83-1.17)	0.85
ADGC/Hollingworth^d^	18,123	18,096				3.6 × 10^-12^
Mayo2/ADGC/Hollingworth^e^	20,661	22,148				1.8 × 10^-13^

Abbreviations: MAF, minor allele frequency; OR, odds ratio for the minor allele; 95% CI, 95% confidence interval

^a^The numbers shown for the series in the Naj *et al*. and Hollingworth *et al*. studies refer to the complete set analyzed. The numbers for the Mayo follow-up data refer to the number of samples successfully genotyped.

^b^MAFs were not reported for LOAD and control groups in the Naj *et al*. or Hollingworth *et al*. studies.

^c^The results shown here for the Mayo2 follow-up dataset combined and for the subseries were obtained using logistic regression adjusted for age, sex and *APOE ε 4 *dosage. The Mayo2 follow-up dataset reported here is independent of that which was incorporated in the GWAS reported by Hollingworth *et al*. The results for each of the Mayo follow-up subseries (Jacksonville, Rochester, Autopsy-confirmed, Norway, Poland and ARUK) are listed immediately below the results for the Mayo2 follow-up dataset combined.

^d^Indicates Fisher's combined p-value for each individual GWAS in the Naj *et al*. study (Combined) and the Hollingworth *et al*. study.

^e^Indicates Fisher's combined p-value for each individual GWAS in the Naj *et al*. study (Combined), the Hollingworth *et al*. study and Mayo2 independent follow-up series.

In order to estimate the overall association of these five variants in our data combined with the previously published associations, we used Fisher's method to combine the p-values for all associations (Table 3; Mayo2/ADGC/Hollingworth). We found that adding our data to those previously reported, increased the strength of evidence for all variants as LOAD risk modifiers (*CD2AP*: p = 6.5 × 10^-11^, *EPHA1*: p = 2.1 × 10^-15^, *ARID5B *rs2588969: p = 2.3 × 10^-9^, *ARID5B *rs4948288: p = 4.0 × 10^-4^, *CD33*: p = 1.8 × 10^-13^).

## Discussion

We report here successful replication of the association of two variants with LOAD in a large (n = 6,835), independent case-control study; rs11767557, which is located 3 kb upstream of *EPHA1 *(p = 5 × 10^-4^) and rs3865444, which is located 373 bp upstream of *CD33 *(p = 0.049). The ORs we observed in our meta-analyses (*EPHA1 *= 0.88, *CD33 *= 0.96) were comparable to those reported by both Naj *et al*. (*EPHA1 *= 0.87, *CD33 *= 0.89) and by Hollingworth *et al*. (*EPHA1 *= 0.90, *CD33 *= 0.89) such that the estimated p-values for association of these variants in all data (n > 42,000) were an impressive 2.1 × 10^-15 ^for *EPHA1 *and 1.8 × 10^-13 ^for *CD33*.

Although our meta-analyses showed successful replication of the association of the *ARID5B *variants rs2588969 (OR = 1.08, p = 0.046) and rs4948288 (OR = 1.11, p = 0.008) with a direction of association consistent with that reported by Hollingworth *et al*. (OR = 1.06 and 1.07, respectively), the associations did not survive adjustment for age-at-diagnosis/entry, sex and *APOE ε 4 *status (p = 0.30 and 0.11, respectively). This covariate-dependent association could explain the opposing association reported by Naj *et al*. in their discovery (OR = 0.88) and replication (OR = 1.05) datasets for rs2588969; the only *ARID5B *variant they tested. Therefore, while estimation of the p-values for association of the *ARID5B *variants in all datasets combined were highly significant (rs2588969; p = 2.3 × 10^-9 ^and rs4948288; p = 4.0 × 10^-4^), interpretation of these associations should be treated with caution and should take into account the age-at-diagnosis/entry, sex and *APOE ε 4 *dosage of the populations. Finally, although the estimated p-value for association of rs9349407 (located in intron 1of *CD2AP*) in all datasets was 6.5 × 10^-11^, there was no evidence for association of this variant in our dataset alone (OR = 0.97, p = 0.56).

Our Mayo2 collection of case-control series studies provided a total of 2,634 LOAD and 4,201 controls. Combining across studies to perform global tests of significance for additive genotypic trend tests gave us 80% power to detect ORs ranging from 1.17 (or 0.855) for variants with a minor allele frequency (MAF) of 0.2 to 1.13 (or 0.883) for variants with a MAF of 0.45 in controls. The study provided approximately 60% power to detect the OR of 1.11 that we report for *CD2AP *(MAF = 0.27).

Case-control studies such as this are not designed to ascertain whether the variants with reported association with LOAD risk are the functional variant but they can identify a linkage disequilibrium (LD) block within which a truly functional variant may reside. Our results indicate that the *EPHA1 *and *CD33 *variants represent excellent candidates for targeted deep sequencing or high density genotyping in order to define the locus further, followed by subsequent functional studies of nearby genes to elucidate the mechanism behind these associations. With the exception of rs9349407, which lies within intron 1of *CD2AP*, all of these variants lie within intergenic regions but for ease of the reader, we have thus far only referred to the nearest gene for each variant. This by no means signifies that these variants (or the functional variants in LD with them) are assumed to affect the expression or function of the nearest gene but may affect other nearby genes. Until it is known which gene underlies these associations, all nearby genes should be included in follow-up functional investigation (all genes that reside within 100 kb of these variants are listed in Additional file 1, Table S1).

## Conclusions

Taken along with our previous publications [5,18,20,21], we have now performed follow-up association studies of 25 of the top GWAS-identified candidate LOAD genes and successfully replicated the association of eleven variants (in or near *ABCA7, BIN1, CD33, CLU, CR1, EPHA1, GAB2, LOC651924, MS4A6A/4E *and *PICALM*), eight of which are currently ranked in the top ten (after *APOE*) on AlzGene. This recent success in replicating genetic association highlights the utility of multiple, large case-control follow-up studies to confirm the novel associations reported by large GWAS, thus confirming them as good candidate genes for functional follow-up studies.

## Methods

### Ethics statement

Approval was obtained from the ethics committee or institutional review board of each institution responsible for the ascertainment and collection of samples. Written informed consent was obtained for all individuals that participated in this study.

### Case-control subjects

The Mayo2 case-control series consisted of Caucasian subjects from the United States ascertained at the Mayo Clinic Jacksonville, Mayo Clinic Rochester, or through the Mayo Clinic Brain Bank. Additional Caucasian subjects from Europe were obtained from Norway [22], Poland [23], and from six research institutes in the United Kingdom that are part of the Alzheimer's Research UK (ARUK) Network. Although the ARUK samples used in this follow-up do not overlap with those employed in the original GWAS publication by Hollingworth *et al*., the same subject/sample ascertainment methodology was followed. The ARUK series included here are from Bristol, Leeds, Manchester, Nottingham, Oxford and Southampton. Since the Manchester cohort only consisted of LOAD cases, the Manchester cases were combined with subjects in the Nottingham series.

### Genotyping

All genotyping was performed at the Mayo Clinic in Jacksonville using TaqMan^® ^SNP Genotyping Assays in an ABI PRISM^® ^7900HT Sequence Detection System with 384-Well Block Module from Applied Biosystems, California, USA. The genotype data was analyzed using the SDS software version 2.2.3 (Applied Biosystems, California, USA).

### Statistical Analyses

Meta-analysis of allelic association and Breslow-Day tests were performed using StatsDirect v2.5.8 software. Meta-analyses were performed using the results from each individual case-control series. Summary ORs and 95% CI were calculated using the DerSimonian and Laird (1986) random-effects model [24]. Breslow-Day tests were used to test for heterogeneity between populations. PLINK software [25] (http://pngu.mgh.harvard.edu/purcell/plink/) was used to perform logistic regression analysis under an additive model adjusting for age-at-diagnosis, sex and *APOE ε *4 dose as covariates. In our analysis of all series combined, series was included as an additional covariate. Since genotype counts were not reported for series included in the Naj *et al*. or Hollingworth *et al*. studies, we employed a Fisher combined test to combine p-values across series. Power calculations, based on a Mantel-Haenszel chi-square test that pooled across six different study groups, were obtained to estimate the detectable odds ratios for an ordinal effect using a range of minor allele frequencies spanning those expected from the candidate variants.

## Abbreviations

ABCA7: ATP-binding cassette, sub-family A (ABC1), member 7; AD: Alzheimer's disease; ADGC: Alzheimer's disease Genetic Consortium; APOE: apolipoprotein E; ARID5B: AT rich interactive domain 5B (MRF1-like); ARUK: Alzheimer's Research United Kingdom; BIN1: bridging integrator 1; Bp: base pair; CD2AP: CD2-associated protein; CD33: CD33 molecule; CI: confidence interval; CLU: clusterin; CR1: complement component (3 b/4 b) receptor 1 (Knops blood group); EPHA1: EPH receptor A1; GAB2: GRB2-associated binding protein 2; GERAD: Genetic and Environmental Risk in Alzheimer's Disease Consortium; GWAS: genome-wide association study; kb: kilobases; LD: linkage disequibrium; LOAD: late-onset Alzheimer's disease; MAF: minor allele frequency; MS4A4A: membrane-spanning 4-domains, subfamily A, member 4; OR: odds ratio; PICALM: phosphatidylinositol binding clathrin assembly protein; SD: standard deviation.

## Competing interests

The authors declare that they have no competing interests.

## Authors' contributions

*Study concept and design: *MMC and SGY. *Sample Collection and Diagnosis*: ARUK, DWD, JOA, MB, NRG-R, RCP, SBS, and ZKW. *Genotyping*: MMC and TAH. *DNA Sample Preparation*: GDB, ML and ZFG. *Analysis and interpretation of data: *JEC, KM, MMC, OB, SGY and VSP. *Drafting of the manuscript: *MMC and OB. *Critical revision of the manuscript for important intellectual content: *KM, MMC, OB, SGY and VSP. *Study supervision: *KM, MMC and SGY. All authors have read and approve the final manuscript.

## Supplementary Material

Additional file 1 **Table S1. Genes located within 100 kb of the five variants tested in this study**. Chr, chromosome. Base pair positions (bp) are relative to the NCBI Human Genome build 36.1. The position of the variant relative to the gene is given as 5' (upstream from the gene's transcription start site) or 3' (downstream from the gene's last exon). Distance indicates the number of base pairs from the variant position to the gene's nearest exon. Click here for file
