# Supplementary material for: Replication of EPHA1 and CD33 associations with late-onset Alzheimer's disease: a multi-centre case-control study
Source: Mol Neurodegener. 2011 Jul 28;6:54. doi: 10.1186/1750-1326-6-54 (PMC3157442; doi:10.1186/1750-1326-6-54)
Supplement: Additional file 1 — Table S1. Genes located within 100 kb of the five variants tested in this study. Chr, chromosome. Base pair positions (bp) are relative to the NCBI Human Genome build 36.1. The position of the variant relative to the gene is given as 5' (upstream from the gene's transcription start site) or 3' (downstream from the gene's last exon). Distance indicates the number of base pairs from the variant position to the gene's nearest exon. [file 1750-1326-6-54-S1.DOC]

**Table S1. Genes located within 100 kb of the five variants tested in this study.** Chr, chromosome. Base pair positions (bp) are relative to the NCBI Human Genome build 36.1. The position of the variant relative to the gene is given as 5’ (upstream from the gene’s transcription start site) or 3’ (downstream from the gene’s last exon). Distance indicates the number of base pairs from the variant position to the gene’s nearest exon.

| **Variant** | **Chr** | **bp** | **Gene** | **Start (bp)** | **End (bp)** | **Relative position of variant** | **Distance (bp)** |
| --- | --- | --- | --- | --- | --- | --- | --- |
| rs9349407 | 6 | 47,561,337 | ***CD2AP*** | 47,553,484 | 47,702,955 | Intron 1 | 0 |
| rs11767557 | 7 | 142,819,261 | ***EPHA1*** | 142,816,107 | 142,798,328 | 5' | 3,154 |
|  |  |  | *ZYXIN* | 142,788,482 | 142,798,324 | 3' | 20,937 |
|  |  |  | *TAS2R60* | 142,850,668 | 142,851,623 | 5' | 32,362 |
|  |  |  | *KIAA0773* | 142,769,967 | 142,760,616 | 5' | 49,294 |
|  |  |  | *TAS2R41* | 142,885,088 | 142,886,011 | 5' | 66,750 |
|  |  |  | *CLCN1* | 142,759,219 | 142,723,341 | 3' | 60,042 |
| rs2588969 | 10 | [63,281,360](http://hapmap.ncbi.nlm.nih.gov/cgi-perl/gbrowse/hapmap28_B36/?name=chr10:63281360..63281360) | ***ARID5B*** | 63,331,449 | 63,526,709 | 5' | 50,089 |
|  |  |  | *LOC219621* | 63,092,725 | 63,196,095 | 3' | 85,265 |
| rs4948288 | 10 | 63,254,539 | ***ARID5B*** | 63,331,449 | 63,526,709 | 5' | 76,910 |
|  |  |  | *LOC219621* | 63,092,725 | 63,196,095 | 3' | 58,444 |
| rs3865444 | 19 | 56,419,774 | ***CD33*** | 56,420,147 | 56,435,086 | 5' | 373 |
|  |  |  | *FLJ40235* | 56,452,776 | 56,464,394 | 5' | 33,002 |
|  |  |  | *SIGLEC7* | 56,337,370 | 56,348,595 | 3' | 71,179 |
|  |  |  | *SIGLEC9* | 56,319,977 | 56,325,378 | 3' | 94,396 |
